# Supplementary material for: Simulating SIR processes on networks using weighted shortest paths
Source: Sci Rep. 2018 Apr 26;8:6562. doi: 10.1038/s41598-018-24648-w (PMC5920074; doi:10.1038/s41598-018-24648-w)
Supplement: Supplementary file 1 — Supplemental Information [file 41598_2018_24648_MOESM1_ESM.pdf]

Supplementary information: Simulating SIR processes on networks using weighted shortest paths

Dijana Tolic[1], Kaj-Kolja Kleineberg[2], and Nino Antulov-Fantulin[2]

[1]Rudjer Bošković Institute, Laboratory for Machine Learning and Knowledge Representations, Zagreb, Croatia
[2]ETH Zürich, Swiss Federal Institute of Technology, COSS, Clausiusstraße 50, 8092 Zurich, Switzerland

Contents

1 Time respecting path equivalence **2**

2 Bond percolation limit equivalence **3**

3 Markov Chain correctness **4**

4 Inference via sampling **10**

5 Approximation details **12**

6 Semi-directed bond percolation **16**

7 Airport network spreading **19**

8 Note on complexity **19**

1 Time respecting path equivalence

The equivalence is shown by the following steps: (i) definition of the general stochastic SIR spreading kinetics; (ii) reduction to process where the random outcomes only depend on the edge weights and (iii) finally reduce the outcome of the process with graph shortest paths.

SIR spreading process:
At the beginning, all nodes are in susceptible state except the source node $i_{(0)}$. Let us denote the indices of nodes that got infected as: $\{i_{(1)}, i_{(2)}, ..., i_{(k)}\}$, where $i_{(1)}$ denotes the index of node that got infected first, $i_{(2)}$ the index of node that got infected second and $i_{(k)}$ as the index of node with k-th infection in the process. Their corresponding times of infection are denoted by $t_{(1)}, t_{(2)}, ..., t_{(k)}$, where $t_{(k)}$ denotes the time when infection of node with index $i_{(k)}$ occurred. Set of susceptible nodes at time $t_{(k)}$ is denoted by set $S(t_{(k)})$. The possible time of infection for some susceptible node $j \in S(t_{(k)})$ from previous infected neighbouring node $t_{(k-1)}$ is

$$t_{i_{(k-1)},j} = t_{(k-1)} + \rho_{i_{(k-1)},j}, \tag{1}$$

where we assign time delay $\rho_{i_{(k-1)},j}$ from conditional distribution $\psi(t|\tau)$ of transmission on recovery time of node $i_{(k-1)}$. Each node has it's own recovery time τ which is sampled from ϕ distribution.

After k infections, the next infection occurs at [9],[10]

$$t_{(k+1)} = \min_{j \in S(t_{(k)})} \min(t_{i_{(0)},j}, t_{i_{(1)},j}, ..., t_{i_{(k)},j}), \tag{2}$$

the minimum transmission time from the infected nodes to the all susceptible node at time $t_{(k)}$.

Reduction to shortest paths:
The transmission time delays $\rho_{m,n}$ on edges are conditionally independent random variables given recovery times τ_m. In that case, we can generate the transmission time delays $\rho_{m,n}$ by preserving the conditional dependence in advance and write them as weights on edges, so process distributions remain unchanged.

When $t_{k+1} = T$ it implies then the shortest path $d(i_{(0)}, i_{(k+1)}) = T$. To see this, we will assume that infection time of source node is $t_{(0)} = 0$. From $t_{k+1} = T$ we conclude that $\min(t_{i_{(0)},i_{k+1}}, t_{i_{(1)},i_{k+1}}, ..., t_{i_{(k)},i_{k+1}}) = T$, where each term can be written as a sum over weights $(\rho_{m_1,n_1}, \rho_{m_2,n_2}, ..., \rho_{m_p,n_p})$ with $m_1 = i_{(0)}, n_p = i_{(k+1)}$. This is done by iterating the recursive relation (1) until we come to the source node where $t_{(0)} = 0$. Now, we interpret the infection times t_k as distances

from the source node $i_{(0)}$ to node $i_{(k)}$ and the distance from the source node to itself is zero. From this, we conclude that

$$\min(t_{i_{(0)},i_{k+1}}, t_{i_{(1)},i_{k+1}}, ..., t_{i_{(k)},i_{k+1}}) = T =$$

$$\min\left\{d(i_{(0)}, i_{(0)}) + \rho_{i_{(0)},i_{k+1}}, d(i_{(0)}, i_{(1)}) + \rho_{i_{(1)},i_{k+1}}, ..., d(i_{(0)}, i_{(k)}) + \rho_{i_{(k)},i_{k+1}}\right\},$$

and there exists no path from $i_{(0)}$ to node $i_{(k+1)}$, which has the total sum along the path less then T. Vice versa, if the shortest path $d(i_{(o)}, i_{k+1}) = T$ it implies that $t_{k+1} = T$.

2 Bond percolation limit equivalence

In this section, we prove that for mean-field mapping by letting t go to infinity, we obtain a realization equivalent to the normal bond percolation realization, to which we refer as **bond percolation limit equivalence:**

$$|S_p(v_i)| = \lim_{t\to\infty} |\{d(v_i, v_k) < t\}| \tag{3}$$

$|S_p(v_i)|$ denotes the size of the set of nodes v_k that are reachable by any finite shortest path length from the source node v_i. The term $|S_p(v_i)|$ denotes the size of the connected bond percolation component with transmissibility p parameter from a source v_i [14].

Next, we show that our mapping establishes links with finite length $\rho_{i,j} < \infty$ with the probability which is equal to the transmissibility p [14]:

$$p = \int_0^\infty \phi(\tau)d\tau \int_0^\tau \psi(t)dt = \int_0^\infty \gamma e^{-\gamma\tau}dt \int_0^\tau \beta e^{-\beta t}dt = \frac{\beta}{\beta+\gamma}. \tag{4}$$

In our mapping, the link with finite length $\rho_{i,j} < \infty$ is formed when $-ln(x)/\beta \leq -ln(y)/\gamma$, for x and y as uniform random numbers $\in [0, 1]$.

$$P(-ln(x)/\beta \leq -ln(y)/\gamma) = P(ln(x) \geq \frac{\beta}{\gamma}ln(y)) = P(x \geq y^{\frac{\beta}{\gamma}}) \tag{5}$$

$$P(x \geq y^{\frac{\beta}{\gamma}}) = \int_{-\infty}^{-\infty} \left(\int_{y^{\frac{\beta}{\gamma}}}^{\infty} f_{x,y}(x,y)dx\right) dy, \tag{6}$$

where $f_{x,y}(x,y)$ is joint density function of variables x and y. As they are independent, $f_{x,y}(x,y) = f_x(x)f_y(y)$. As both x and y are uniform random numbers $\in [0,1]$, their densities are $f_x(x) = \mathbf{1}_{[0,1]}$ and $f_y(y) = \mathbf{1}_{[0,1]}$.

$$P(x \geq y^{\frac{\beta}{\gamma}}) = \int_{-\infty}^{-\infty} \mathbf{1}_{[0,1]}(y) \left(\int_{y^{\frac{\beta}{\gamma}}}^{\infty} \mathbf{1}_{[0,1]}(x) dx \right) dy = \int_0^1 \left(\int_{y^{\frac{\beta}{\gamma}}}^{1} dx \right) dy = \frac{\beta}{\beta + \gamma} \tag{7}$$

which is equal to transmissibility parameter p [14]. Therefore, we conclude that both bond percolation and time augmented bond percolation are the equivalent stochastic processes for the formation of edges when the finite edge weights are taken into account.

3 Markov Chain correctness

We are interested in Markov Chain with state space: $\Omega = \{\mathcal{G}_1, \mathcal{G}_2, ..., \mathcal{G}_i, ...\}$, with transitions $P(\mathcal{G}_i \to \mathcal{G}_j)$ between weighted graphs.

Note that graphs $\mathcal{G}$ have continuous non-negative real weights and that the density function for weight ρ of a specific link is $\int_0^\infty f(\tau,\rho)d\tau$, where $f(\tau,\rho)$ is the joint density for recovery time τ and transmission time ρ:

$$f(\tau,\rho) = \phi(\tau) \left(\psi(\rho) \mathbf{1}_{[0,\tau\rangle}(\rho) + \delta(\rho - \infty) \int_\tau^\infty d\rho \psi(\rho) \right),$$

where $\mathbf{1}_{[0,\tau\rangle}(\rho)$ is the identity function which is equal to one on a range $[0,\tau\rangle$ and $\delta(t-\infty)$ is the Dirac delta distribution. Before proceeding, we will use the discrete approximation of the non-negative weights i.e. density functions $\phi(\tau)$, $\psi(\rho)$, $f(\tau,\rho)$ become probability mass functions $g_\phi(\tau)$, $g_\psi(\rho)$, $g_{\phi,\psi}(\tau,\rho)$. This approximation essentially tells us that we use the finite number of possible non-negative weights to approximate density distributions, which is the case for all numeric simulations of continuous functions. This enables us to use the formalism of Markov Chain for discrete state space.

By design, the Markov Chain has stationary distribution $\mathcal{P}(\mathcal{G})$, which is equal to the probability that this graph is generated by our mapping:

$$P(\mathcal{G}) = \prod_{A_{i,j}=1} g_{\psi|\phi}(\rho_{i,j}|\tau_{i,j}) g_\phi(\tau_{i,j}). \tag{8}$$

The probability of a particular instance weighted graph due to the independence is a product of probabilities over weights on edges generated by our mapping. The probability of having a weight $\tau_{i,j}$ is $g_\phi(\tau_{i,j})$ and probability of having a weight $\rho_{i,j}$ for a given $\tau_{i,j}$ is $g_{\psi|\phi}(\rho_{i,j}|\tau_{i,j})$.

Convergence to stationary state

The existence of stationary distribution of the Markov Chain in discrete time and discrete states is guaranteed by detailed balance property and the uniqueness by ergodicity.

First, we show that the detailed balance and ergodicity property holds for mean-field mapping and later generalize for exact mapping.

We show why the **detailed balance** holds if we choose the transition $P(\mathcal{G}_i \to \mathcal{G}_j)$ such that the weight of the randomly selected edge (i^*, j^*) is changed by generating new weight with our mapping.

We write the detailed balance condition:

$$P(\mathcal{G}_i)P(\mathcal{G}_i \to \mathcal{G}_j) = P(\mathcal{G}_j)P(\mathcal{G}_j \to \mathcal{G}_i). \tag{9}$$

The probability of generating the particular graph $\mathcal{G}_i$ is:

$$P(\mathcal{G}_i) = \prod_{A_{k,l}=1} g_{\psi|\phi}(\rho_{k,l}|\tau_{k,l})g_\phi(\tau_{k,l}), \tag{10}$$

where $\rho^i_{k,l}$ denotes the transmission weight on edge (k,l) in $\mathcal{G}_i$ and $\tau^i_{k,l}$ denotes the recovery time for edge (k,l) in $\mathcal{G}_i$. Similarly, for graph $\mathcal{G}_j$ we can write the probability $P(\mathcal{G}_j)$. Now, the transition between $\mathcal{G}_i$ and $\mathcal{G}_j$ happens by selecting the random edge (m^*, n^*) with uniform probability c and changing the weight on it.

$$P(\mathcal{G}_i \to \mathcal{G}_j) = cg_\phi(\tau^j_{m^*,n^*})g_{\psi|\phi}(\rho^j_{m^*,n^*}|\tau^j_{m^*,n^*}). \tag{11}$$

We can extract the edge (m^*, n^*) in $\mathcal{G}_i$ from equation (10):

$$P(\mathcal{G}_i) = g_\phi(\tau^i_{m^*,n^*})g_{\psi|\phi}(\rho^i_{m^*,n^*}|\tau^i_{m^*,n^*}) \prod_{A^i_{k,l}=1:(k,l)\neq(m^*,n^*)} g_{\psi|\phi}(\rho^i_{k,l}|\tau^i_{k,l})g_\phi(\tau^i_{k,l}) \tag{12}$$

and similarly for graph $\mathcal{G}_j$:

$$P(\mathcal{G}_j) = g_\phi(\tau^j_{m^*,n^*})g_{\psi|\phi}(\rho^j_{m^*,n^*}|\tau^j_{m^*,n^*}) \prod_{A^j_{k,l}=1:(k,l)\neq(m^*,n^*)} g_{\psi|\phi}(\rho^j_{k,l}|\tau^i_{k,l})g_\phi(\tau^j_{k,l}). \tag{13}$$

Now, the ratio $\frac{P(\mathcal{G}_i)}{P(\mathcal{G}_j)}$ is

$$\frac{g_\phi(\tau^i_{m^*,n^*})g_{\psi|\phi}(\rho^i_{m^*,n^*}|\tau^i_{m^*,n^*}) \prod_{A^i_{k,l}=1:(k,l)\neq(m^*,n^*)} g_{\psi|\phi}(\rho^i_{k,l}|\tau^i_{k,l})g_\phi(\tau^i_{k,l})}{g_\phi(\tau^j_{m^*,n^*})g_{\psi|\phi}(\rho^j_{m^*,n^*}|\tau^j_{m^*,n^*}) \prod_{A^j_{k,l}=1:(k,l)\neq(m^*,n^*)} g_{\psi|\phi}(\rho^j_{k,l}|\tau^i_{k,l})g_\phi(\tau^j_{k,l})} = \tag{14}$$

$$= \frac{g_\phi(\tau^i_{m^*,n^*}) g_{\psi|\phi}(\rho^i_{m^*,n^*}|\tau^i_{m^*,n^*})}{g_\phi(\tau^j_{m*}) g_{\psi|\phi}(\rho^j_{m^*,n^*}|\tau^j_{m^*})}$$

since the graphs $\mathcal{G}_i$ and $\mathcal{G}_j$ share all the weights except on the randomly selected edge (m^*, n^*). Next, we see that this ratio is equal to the ratio of transitions rates:

$$\frac{g_\phi(\tau^i_{m^*,n^*}) g_{\psi|\phi}(\rho^i_{m^*,n^*}|\tau^i_{m^*,n^*})}{g_\phi(\tau^j_{m^*,n^*}) g_{\psi|\phi}(\rho^j_{m^*,n^*}|\tau^j_{m^*,n^*})} = \frac{P(\mathcal{G}_j \to \mathcal{G}_i)}{P(\mathcal{G}_i \to \mathcal{G}_j)} \tag{15}$$

and we conclude that the detailed balance holds.

The second thing that needs to be proved is **ergodicity**. We know, that Markov Chain is ergodic if it is irreducible, aperiodic and positive recurrent. A finite state Markov chain is irreducible and positive recurrent if there's a finite number N such that any state can be reached from any other state in exactly N steps. To prove this, we consider two generic states $\mathcal{G}_i$ and $\mathcal{G}_j$ and show that there is a positive probability for reaching state $\mathcal{G}_j$ from state $\mathcal{G}_i$ in finite number of steps. Any state $\mathcal{G}_j$ can be reached from state $\mathcal{G}_i$ with the transition path $\mathcal{G}_0 \to \mathcal{G}_1 \to ... \to \mathcal{G}_N$, where $\mathcal{G}_0 = \mathcal{G}_i$ and $\mathcal{G}_N = \mathcal{G}_j$, where weights are changed link by link. Since number of distinct link in network is finite so it is the number of transitions N. As the transitions are independent the probability $P(\mathcal{G}_i \to \mathcal{G}_j)$ is equal to the product $\prod_{k=0}^{N-1} P(\mathcal{G}_k \to \mathcal{G}_{k+1})$, which is positive since every $P(\mathcal{G}_k \to \mathcal{G}_{k+1})$ is positive. Every product is positive as the weights in $\mathcal{G}$ come from the distributions ψ and ϕ. As the probability $P(\mathcal{G}_i \to \mathcal{G}_i) > 0$ that means that the Markov Chain is also aperiodic.

Next, we show minimum changes that are needed so that the proofs hold for the **exact mapping**. The transitions in the exact mapping are done by updating the weights of the first neighbourhood of a randomly selected node by re-sampling $\psi(\cdot)$ and $\phi(\cdot)$ functions.

Lets denote the randomly selected node with index $m*$. Then the transition is written as:

$$P(\mathcal{G}_i \to \mathcal{G}_j) = c \prod_{A_{m*,l}=1} g_{\psi|\phi}(\rho^j_{m*,l}|\tau^j_{m*,l}) g_\phi(\tau^j_{m*,l}). \tag{16}$$

Then, by analogy to the initial proof, we see that this ratio

$$\frac{P(\mathcal{G}_i)}{P(\mathcal{G}_j)} = \frac{\prod_{A_{m*,l}=1} g_{\psi|\phi}(\rho^i_{m*,l}|\tau^i_{m*,l}) g_\phi(\tau^i_{m*,l})}{\prod_{A_{m*,l}=1} g_{\psi|\phi}(\rho^j_{m*,l}|\tau^j_{m*,l}) g_\phi(\tau^j_{m*,l})} = \frac{P(\mathcal{G}_j \to \mathcal{G}_i)}{P(\mathcal{G}_i \to \mathcal{G}_j)} \tag{17}$$

which implies that the detailed balance holds.

Similarly, like in the proof for the ergodicity for the mean-field mapping, the ergodicity holds for the exact mapping. The only difference is that the weights are changed neighbourhood by neighbourhood. Since number of distinct neighbourhoods is equal to the number of nodes in the network the number of transitions N is always finite. And by analogy $P(\mathcal{G}_i \to \mathcal{G}_j)$ is equal to the product $\prod_{k=0}^{N-1} P(\mathcal{G}_k \to \mathcal{G}_{k+1})$, which is positive since every $P(\mathcal{G}_k \to \mathcal{G}_{k+1})$ is positive.

Relation to Gibbs sampling

First, we consider more general Metropolis-Hastings sampling and then show why we have a rejection free algorithm. Metropolis-Hestings method separate the transition

$$P(\mathcal{G}_i \to \mathcal{G}_j) = g(\mathcal{G}_i \to \mathcal{G}_j) A(\mathcal{G}_i \to \mathcal{G}_j), \tag{18}$$

in two sub-steps: the proposal $g(\mathcal{G}_i \to \mathcal{G}_j)$ of next move and the acceptance-rejection $A(\mathcal{G}_i \to \mathcal{G}_j)$. The acceptance-rejection rule is derived by inserting the acceptance-rejection (18) into detailed balance (9):

$$\frac{A(\mathcal{G}_i \to \mathcal{G}_j)}{A(\mathcal{G}_j \to \mathcal{G}_i)} = \frac{P(\mathcal{G}_j) g(\mathcal{G}_j \to \mathcal{G}_i)}{P(\mathcal{G}_i) g(\mathcal{G}_i \to \mathcal{G}_j)} \tag{19}$$

and the acceptance rule that meets the condition above is the following:

$$A(\mathcal{G}_i \to \mathcal{G}_j) = \min\left(1, \frac{P(\mathcal{G}_j) g(\mathcal{G}_j \to \mathcal{G}_i)}{P(\mathcal{G}_i) g(\mathcal{G}_i \to \mathcal{G}_j)}\right), \tag{20}$$

this can be verified quickly with two possibilities only either the $P(\mathcal{G}_j) g(\mathcal{G}_j \to \mathcal{G}_i)$ is grater or lower than $P(\mathcal{G}_j) g(\mathcal{G}_j \to \mathcal{G}_i)$.

If the proposal rule for transitions $g(\mathcal{G}_i \to \mathcal{G}_j) = g(\mathcal{G}_j \to \mathcal{G}_i)$ is symmetric we get the Metropolis algorithm with acceptance rule: $A(\mathcal{G}_i \to \mathcal{G}_j) = min(1, P(\mathcal{G}_j)/P(\mathcal{G}_i))$.

If the proposal rule for transitions $g(\mathcal{G}_i \to \mathcal{G}_j) = P(\mathcal{G}_i \to \mathcal{G}_j)$ is conditional probability then we get the $A(\mathcal{G}_i \to \mathcal{G}_j) = 1$ rejection-free sampler (Gibbs sampler).

$$A(\mathcal{G}_i \to \mathcal{G}_j) = \min\left(1, \frac{P(\mathcal{G}_j) P(\mathcal{G}_j \to \mathcal{G}_i)}{P(\mathcal{G}_i) P(\mathcal{G}_i \to \mathcal{G}_j)}\right) = \min\left(1, \frac{P(\mathcal{G}_j, \mathcal{G}_i)}{P(\mathcal{G}_i, \mathcal{G}_j)}\right) = 1 \tag{21}$$

In our case, the transitions between weighted graphs $P(\mathcal{G}_i \to \mathcal{G}_j)$ are constructed by changing the weights on the randomly selected edge and by re-sampling them from ψ and ϕ. In principle, we could also do the Metropolis-Hastings update by selecting other proposal functions $g(\mathcal{G}_i \to \mathcal{G}_j)$, but the Gibbs sampling gives us the advantage that we do not need to evaluate the probability of a graph (10) explicitly, we get the rejection free sampler and we just need to resample the new time delays directly form ϕ, ψ.

The existence of stationary distribution of this Markov Chain is guaranteed by detailed balance property and the uniqueness by ergodicity. And finally, from each sampled graph the dynamics is obtained when we choose the initial conditions (source nodes) and is governed by the network paths.

In Figure 1, we compare the accuracy of the estimated expected outbreak size for the SIR dynamics on the regular lattice network. As a ground truth, we use the kinetic Monte Carlo simulation [5] and compare the accuracy with our time augmented bond percolation mapping estimations. As the number of simulations increases, we obtain results more accurate than the Dynamic Message Passing algorithm [11], which makes large errors due to the existence of loops in the network.

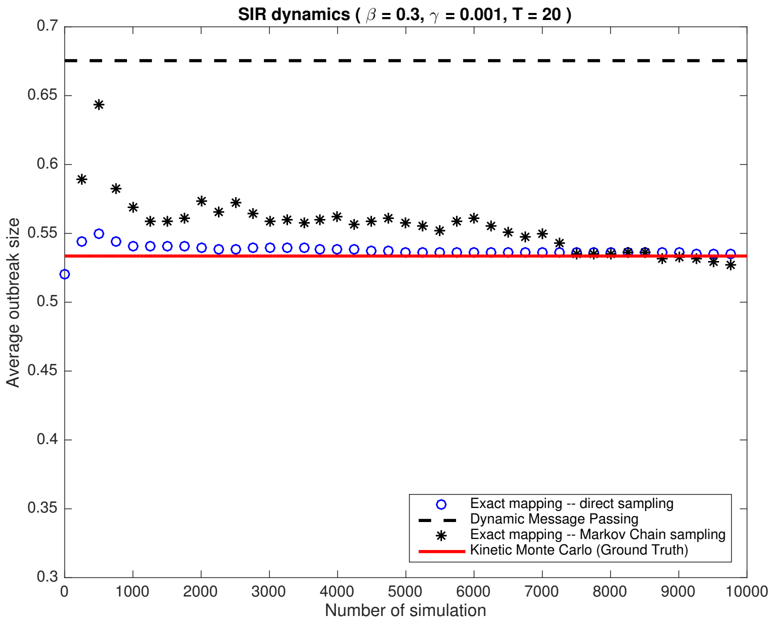

Figure 1: Final outbreak size averaged over different number of simulations for the SIR dynamics transmission rate $\beta = 0.3$, recovery rate $\gamma = 0.001$ and snapshot time $T = 20$ (discrete time) on a 4-connected two dimensional regular lattice (10^3 nodes). Comparison of different models: (i) red line is a kinetic Monte Carlo estimation (ground truth) with 10^4 simulations [5], (ii) black doted line is a Dynamic Message Passing (Belief propagation) estimation [11], (iii) blue circle markers is our proposed mapping with direct sampling estimation and (iv) black star markers is our proposed mapping estimation with Markov Chain. Our estimations outperform Dynamic Message Passing estimations due to the existence of loops in a network.

4 Inference via sampling

Now we state the following theorem from Markov Chain theory [13].
Theorem Let $\{\mathcal{G}_1, ..., \mathcal{G}_n\}$ be random variables distributed according to the Markov Chain that satisfies the detailed balance and ergodic property with initial condition $\mathcal{G}_0$ and let $f(\mathcal{G})$ be any real valued scalar function, then the following holds:

- The probability distribution $\mathcal{G}_n$ converges to the stationary distribution:

$$\lim_{n\to\infty} P(\mathcal{G}_n = \mathcal{G}) = P(\mathcal{G})$$

- Time average converges to the average over stationary distribution almost surely

$$\langle f(\mathcal{G})\rangle = \lim_{n\to\infty} \frac{1}{n}\sum_{i=1}^{n} f(\mathcal{G}_i) = \sum_{\mathcal{G}} P(\mathcal{G}) f(\mathcal{G}),$$

  where $f(\mathcal{G})$ function is used for estimation of different properties over graph $\mathcal{G}$.

We list different estimation functions that can be used with time augmented bond percolation mapping:

- Expected total outbreak size from node v_i can be estimated with the following function:

$$f(\mathcal{G}, v_i) = \sum_j \Theta^{'}(\infty - d(v_i, v_j)),$$

  where $\Theta^{'}(x)$ is the Heaviside step function, which equals to 1 when $x > 0$ i.e. $d(v_i, v_j) < \infty$ and 0 otherwise.

- Expected evolution of number of infected nodes at time t from node v_i can be estimated with the following function:

$$f(\mathcal{G}, v_i, t) = \sum_j \Theta(t - d(v_i, v_j)).$$

  where $\Theta(x)$ is the Heaviside step function, which equals to 1 when $x \geq 0$ i.e. $t \geq d(v_i, v_j)$ and 0 otherwise.

- The expected distance time for the spreading to propagate from node v_i to node v_j can be estimated with the following function:

$$f(\mathcal{G}, v_i, v_j) = d_{\mathcal{G}}(v_i, v_j)$$

- The estimation of microscopic configuration e.g. probability of specific realization described by the set of infected nodes R_* (observation) from specific source node v_i can be estimated with kernel density estimator [2, 12]:

$$f(\mathcal{G}, R_*) = e^{\frac{-(\varphi(\mathcal{G},R_*)-1)^2}{a^2}},$$

  where $\varphi(\mathcal{G}, R_*)$ is the similarity function between specific realization R_* and estimated realization from weighted graph $\mathcal{G}$ from source node v_i and a is the kernel width parameter.

$$\varphi(\mathcal{G}, R_*) = \frac{1}{N} \sum_{j \in R_*} \Theta(t - d(v_i, v_j)) + \frac{1}{N} \sum_{j \in R_*^c} \Theta^{'}(d(v_i, v_j) - t),$$

  where R_*^c denotes the complement i.e. set of non-infected nodes and N is the normalization constant i.e. total number of nodes in a network. Function $\Theta(x)$ equals to 1 when $t \geq d(v_i, v_j)$ and 0 otherwise. Function $\Theta^{'}(x)$ equals to 1 when $d(v_i, v_j) > t$ and 0 otherwise.

- Estimations based on averages of i.i.d. samples have convergence rate $O(n^{-1/2})$, where the Berry-Esseen theorem gives bounds on the constants for convergence. Main advantage of using the kernel estimators [12] is that they have a faster convergence rate. They have physical analogy to using the information potential field to non-parametrically estimate realization probability densities.

- Finally, we do not need to set the Kernel width parameter a in advance. We can choose the parameter a as the infimum of the set of parameters for which the estimations have converged [2].

5 Approximation details

Let us recall the mapping from the main paper. In particular, a *time-respecting weighted network* instance $\mathcal{G}_k$ is created by taking the input network G and assigning weights to the edges with the Inverse Smirnov transform:

$$\rho_{i,j} = \begin{cases} \Psi^{-1}(x): & \Psi^{-1}(x) \leq \Phi^{-1}(y), \\ \infty: & \Psi^{-1}(x) > \Phi^{-1}(y), \end{cases} \tag{22}$$

where x and y are uniform random numbers $\in [0,1]$, $\Phi^{-1}(x)$ and $\Psi^{-1}(y)$ are inverse functions of the cumulative inter-event distributions: $\Phi(t) = \int_0^t dt'\phi(t')$ and $\Psi(t) = \int_0^t dt'\psi(t')$. The quantities $\Psi^{-1}(y)$ and $\Phi^{-1}(x)$ respectively represent the samples of the transmission and recovery time obtained with the Inverse Smirnov transform of inter-event distributions.

The mapping can be done in two ways:

- (a) **exact mapping** is obtained by generating a random variable y for each node and variable x for each edge. This takes correlation among neighbours into the account and generates the ensemble of **directed weighted networks**. In a limit of time this leads to the semi-directed bond percolation networks [10].
- (b) **mean-field mapping** is obtained by generating random variables x and y per each edge. This assumes the independence i.e. mean-field approximation, which is accurate when $\beta >> \gamma$ and generates the ensemble of **undirected weighted networks**. In a limit of time this leads to the bond percolation networks.

Under the assumption that the transmission rate is much larger than recovery rate ($\beta/\gamma \gg 1$), the transmission time delays $\rho_{m,n}$ on edges are independent variables and the simplified mapping is exact. In Figure 2 of this response letter, we show the accuracy of the mapping for different values of β/γ. When $\beta \approx \gamma$, the transmission time delays $\rho_{m,n}$ on edges are conditionally independent of recovery time and no exact bond percolation mapping exists. Contrary to simple mean-field mapping, the exact mapping makes statistically small errors in estimating the outbreak size. In order to provide experimental evidence that the exact mapping is exact, we show that the estimation errors converge to zeros as number of samples are increased. The relative error is measured towards the outbreak size estimated with the Kinetic Monte Carlo simulation. In the next answer, we show more formal theoretical evidences for capturing dynamical correlations among neighbours.

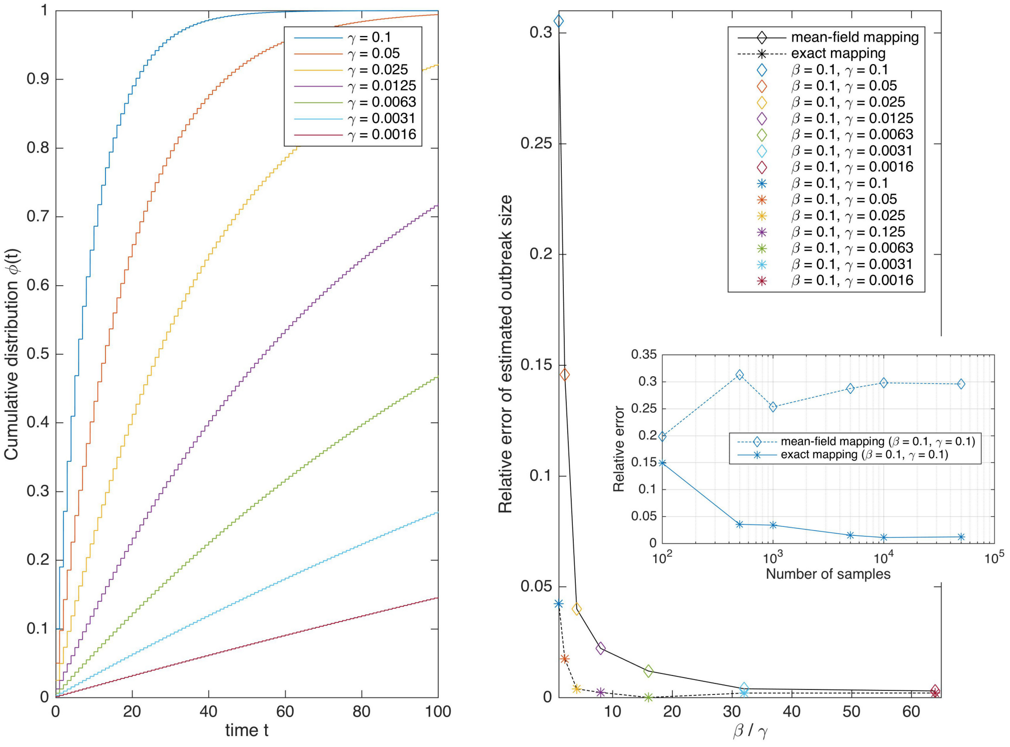

Figure 2: Approximation errors for mean-field mapping and exact mapping for different parameters β/γ for SIR (β, γ) discrete time process. **Left**: Cumulative density function $\phi(t) = \sum_{T=1}^{\infty} \delta(t-T)\gamma(1-\gamma)^{T-1}$ for recovery events with different γ rates. The transmission density distribution function $\psi(t) = \sum_{T=1}^{\infty} \delta(t-T)\beta(1-\beta)^{T-1}$ has fixed transmission rate $\beta = 0.1$. **Right**: Relative error of estimating the total outbreak size with our method versus the kinetic Monte Carlo ("ground truth"), which is a statistical exact representation of the process [4, 3]. Results were obtained on a 4-connected two dimensional regular lattice (10^2 nodes) .

Next, we demonstrate that our mapping is taking all possible paths between source and destination nodes into the account. Consider a simple network model of n_c chains of length l, such that the source node s is connected to the starting node of every chain and the destination node d is connected to the end of every chain.

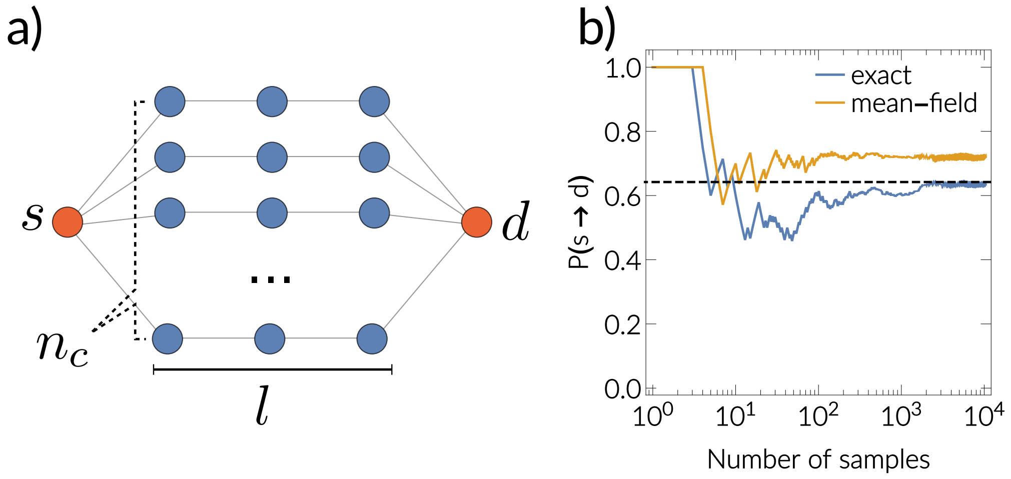

Figure 3: **(a)** Sketch of simple Network model of n_c chains with l nodes. **(b)** Experiment on network model with $n = 20$, $l = 3$ with Poisson SIR ($\beta = 1, \gamma = 1$). The blue curve represents the estimations with the exact mapping (c.i.d. case) and the red curve with the mean-field mapping i.i.d. case)

The probability that a node s infects node d with the SIR dynamics $(\phi(t), \psi(t))$ is:

$$P(s \to d) = 1 - \sum_{j=0}^{n_c} p_{n,j} \cdot \left(1 - p_{1,1}^{l}\right)^{j} \tag{23}$$

where $p_{n,k}$ denotes the probability that node with n neighbours infects k of them. The term $\left(1 - p_{1,1}^{l}\right)$ denotes the probability that infection does not spread though a chain with l nodes to the destination node. Then term $\left(1 - p_{1,1}^{l}\right)^{j}$ denotes the probability that the infection does not spread though any of j chains. Finally, the term $\sum_{j=0}^{n}(\cdot)$ sums all possible contributions of how many chains have been infected from source node s.

$$p_{n,k} = \int_0^{\infty} \phi(\tau) d\tau \binom{n}{k} (1 - \Psi(\tau))^{(n-k)} \, \Psi(\tau)^{k}, \tag{24}$$

where $\Psi(\tau) = \int_0^\tau \psi(t)dt$. For Poissionan process this becomes

$$p_{n,k} = \binom{n}{k} \frac{\gamma}{\beta} \frac{\Gamma(k+1)\Gamma(\frac{\gamma+\beta(n-k)}{\beta})}{\Gamma(k+1+\frac{\gamma+\beta(n-k)}{\beta})}. \tag{25}$$

The estimations with the mappings are done by generating n weighted graphs $\{\mathcal{G}_1, ..., \mathcal{G}_n\}$ and obtaining the following estimate:

$$\hat{P}(s \to d) = \frac{1}{n} \sum_{i=1}^{n} 1(d_{\mathcal{G}_i}(s,d) < \infty). \tag{26}$$

The convergence rate of estimation $\hat{P}(s \to d)$ based on n independent samples is bounded with the BerryEsseen inequality as $O(n^{-1/2})$. In Fig. 3, we demonstrate that the estimations converge to the analytical solution on a toy network model. This confirms that the shortest paths in the ensemble of weighted networks are taking into the account stochastic spreading along all possible paths in the original unweighted network. Next in Fig. 4, we show that the exact mapping converges to the analytical solution even on a large networks, constructed with the procedure from Fig. 3 with $n_c = 62500$ chains of length $l = 16$, such that we obtain total of $N = 10^6$ nodes.

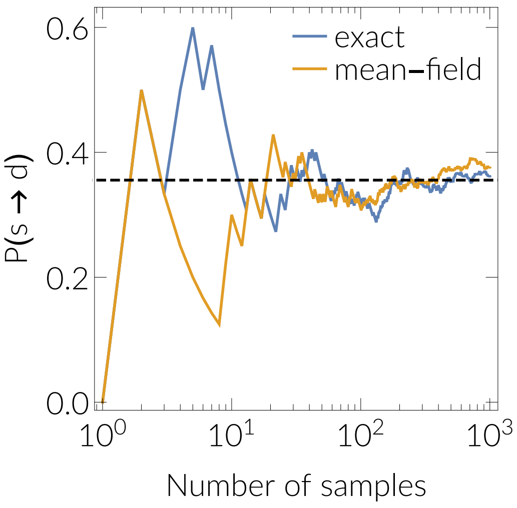

Figure 4: Convergence of exact mapping to the analytical solution on a network with $N = 10^6$ nodes, $n_c = 62500$ chains with length $l = 16$.

6 Semi-directed bond percolation

Previously, the SIR dynamics without memory has been mapped [10] to the semi-directed bond percolation networks. However, still this mapping was only describing the asymptotic dynamics ($t = \infty$). In this section, we formalize this mapping with generalization of transmissibility from the link level to the neighbourhood level in order to make connection with the semi-directed bond percolation. Furthermore, we show that in our mapping we only need to change small adjustments so that it becomes exact mapping with weights.

Newman transmissibility for the link

Let us look at the continuous time SIR model, where infected node transmits the disease to susceptible node at an average rate β and infected nodes recover at the constant rate γ. So the probability of recovering in any short time interval dt is γdt and the probability of transmitting the disease in any short time interval dt is βdt.

The probability of recovering in any short time interval dt is γdt and the probability that the node is still infected after a τ time is:

$$\lim_{dt\to 0}(1-\gamma dt)^{\frac{\tau}{dt}} = \lim_{\frac{\tau}{dt}\to\infty}(1+\frac{-\gamma\tau}{\frac{\tau}{dt}})^{\frac{\tau}{dt}} = e^{-\gamma\tau}, \tag{27}$$

and the probability that the node remains infected this long and then recovers in the interval $\tau + \mathrm{d}\tau$ is: $e^{-\gamma\tau}\gamma\mathrm{d}\tau$, which is a standard formulation waiting times is probability density function $\phi(\tau) = \gamma e^{-\gamma\tau}$ for the exponential distribution with parameter γ. Now, we can use the same analogy and say that the waiting time for transmission when there is no recovery to stop it is the probability density function $\psi(t) = \beta e^{-\beta t}$. Note, that the transmission through edge can happen only prior to the recovery of node at time τ which is described by the pdf $\phi(\tau)$,. Then the total probability of transmission through edge is:

$$T = \int_0^\infty \phi(\tau)d\tau \int_0^\tau \psi(t)dt = \int_0^\infty \gamma e^{-\gamma\tau}dt \int_0^\tau \beta e^{-\beta t}dt = 1 - \frac{\gamma}{\beta+\gamma}. \tag{28}$$

Exact transmissibility for the neighbourhood

Consider the focal node with n neighbours, the transmissibility for the first neighbourhood (with all correlations) or more precisely the probability $T_{n,k}$ that k out of n nodes get infected prior to the recovery time of central node is:

$$T_{n,k} = \binom{n}{k}\frac{\gamma}{\beta}\frac{\Gamma(k+1)\Gamma(\frac{\gamma+\beta(n-k)}{\beta})}{\Gamma(k+1+\frac{\gamma+\beta(n-k)}{\beta})}. \tag{29}$$

Proof
If we know the recovery time τ of central node, the conditional probability of transmission through any edge is $\int_0^\tau \psi(t)dt = 1 - e^{-\beta t}$.

Now, as we have n nodes the probability that k nodes gets infected if the recovery time of central node is τ is:

$$P_{(n,k)|\tau} = \binom{n}{k} e^{(-\beta t)(n-k)}(1-e^{-\beta t})^k, \tag{30}$$

where $(1-e^{-\beta t})^k$ describes that through k transmission passes and through $n-k$ does not passes $e^{(-\beta t)(n-k)}$ and there are $\binom{n}{k}$ different combinations of k nodes.

Then, we need to to get the joint distribution $P_{(n,k),\tau} = P_{(n,k)|\tau}P_\tau$:

$$P_{(n,k),\tau} = \gamma e^{-\gamma\tau}\binom{n}{k} e^{(-\beta t)(n-k)}(1-e^{-\beta t})^k. \tag{31}$$

And finally, we integrate over recovery time $\int_0^{+\infty} P_{(n,k),\tau}\mathrm{d}\tau$ to loose dependence on τ:

$$P_{n,k} = \int_0^{+\infty} \gamma e^{-\gamma\tau}\binom{n}{k} e^{(-\beta t)(n-k)}(1-e^{-\beta t})^k \mathrm{d}\tau. \tag{32}$$

After integration we get the following expression:

$$T_{n,k} = \binom{n}{k}\frac{\gamma}{\beta}\frac{\Gamma(k+1)\Gamma(\frac{\gamma+\beta(n-k)}{\beta})}{\Gamma(k+1+\frac{\gamma+\beta(n-k)}{\beta})}. \tag{33}$$

The $T_{n.k}$ is a valid distribution: (1) $\sum_k T_{n.k} = 1$ and (2) $T_{n.k} \geq 0$ (all arguments to the gamma functions are real and non-negative).

Relation to Newman transmissibility and bond percolation

Now, we show that neighbourhood transmissibility $T_{n,k}$ is equal to Newman transmissibility [14] only for $T_{n=1,k=1}$. But, first let us calculate $T_{n,0}$:

$$T_{n,0} = \frac{\gamma}{\beta}\frac{\Gamma(\frac{\gamma+n\beta}{\beta})}{\Gamma(1+\frac{\gamma+n\beta}{\beta})} = \frac{\gamma}{\beta}\frac{\Gamma(\frac{\gamma}{\beta}+n)}{\Gamma(\frac{\gamma}{\beta}+n+1)}, \tag{34}$$

now by the property of Gamma function $\Gamma(t+1) = t\Gamma(t)$ we obtain the following:

$$T_{n,0} = \frac{\gamma}{\beta}\frac{\Gamma(\frac{\gamma}{\beta}+n)}{(\frac{\gamma}{\beta}+n)\Gamma(\frac{\gamma}{\beta}+n)} = \frac{\gamma}{\gamma+n\beta}. \tag{35}$$

Note, that thus $T_{n=1,k=1} + T_{n=1,k=0} = 1$ (transmission either happens or does not happens) and therefore $T_{n=1,k=1} = 1 - T_{n,0} = 1 - \frac{\gamma}{\gamma+\beta} = T$.

For general distributions $\phi(\cdot)$ and $\psi(\cdot)$, the generalized transmissibility for the neighbourhood is:

$$T_{n,k} = \int_0^\infty \phi(\tau) d\tau \binom{n}{k} (1 - \Psi(\tau))^{(n-k)} \Psi(\tau)^k, \tag{36}$$

where $\Psi(\tau) = \int_0^\tau \psi(t)dt$. For $n = k = 1$, we get the standard transmissibility

$$T_{n=1,k=1} = \int_0^\infty \phi(\tau) d\tau \Psi(\tau) = \int_0^\infty \phi(\tau) d\tau \int_0^\tau \psi(t) dt = T. \tag{37}$$

7 Airport network spreading

In order to generate the effective distance matrix on the world airport transportation network, we use the world airport transportation system data [1] [19, 6]. Model of spreading is a simple model of diffusion of the infected agents along the airport transportation network. Each node represents an airport and each edge represents a connection where the diffusion of infected and susceptible travellers happen. From the flux data between nodes, we estimate the corresponding transmission rate per flight $\beta_{i,j}$ for every link in the network. The rate is now the estimated ratio of number of passengers traveling from node v_i to node v_j and the total outgoing traffic i.e. $\beta_{i,j} = F_{ij}/\sum_k F_{i,k}$. Here, we model a discrete time SIR spreading process, where $\beta_{i,j}$ is estimated from flux travel data. The recovery rate $\gamma \approx 0$, which describes rapid pandemic spreading, where infected airport can not fully get recovered in short time scale. Then the corresponding transmission inter-event density distribution is $\psi(t) = \sum_{T=1}^{\infty} \delta(t-T)\beta(1-\beta)^{T-1}$ i.e. geometric distribution – discrete analog of exponential distribution and $\phi(t) = \delta(t-\infty)$, where $\delta(t)$ denotes the Dirac delta distribution. Essentially, the weights on the edges are samples of geometric distribution, whose rate parameter is estimated from data.

8 Note on complexity

The running time of the mapping algorithm estimations from single source scales as $O(E + N\log N)$, where N is the number of nodes and E is the number of edges in a network. In Fig. 5 and Fig. 6, we explicitly show the running time for the networks up to 10^6 nodes. As shown in the figure, the running time from one source to all nodes is only a few seconds for 10^6 nodes.

However, in case that we need to make estimations from all posible sources, one can use dynamic shortest paths algorithms [8] and the weights are changed with the proposed Markov Chain transitions. The sampling of edge weight has constant time complexity and the dynamically recalculation of shortest paths has complexity of $O(N^2log^3(N))$ [7]. Note, that state-of-the-art algorithms can even improve the complexity of updating all–to–all shortest paths after each edge weight update. Dynamically recalculation of the shortest paths for dense networks with N nodes and E edges can be done in $\mathrm{O}\big(N^2(\log N + \log^2((N+E)/N))\big)$ [16] amortized time. For sparse networks with N nodes and E edges the dynamically recalculation of the shortest paths can be done in $\mathrm{O}\big(N^2\log\log N + NE\big)$ [15]. Furthermore, for efficient approximation techniques for computation of shortest paths see [18, 17, 1].

[1] Official Airline Guide, http://www.oag.com

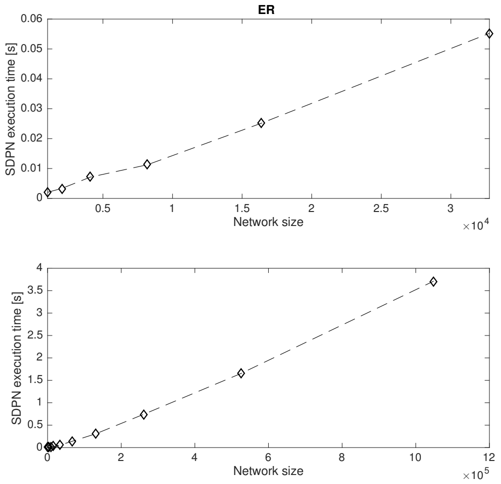

Figure 5: Run-time experiment of the single source mapping algorithm for Erdos–Renyi (ER) network model with sizes from $2^{10} \approx 10^3$ to $2^{20} \approx 10^6$.

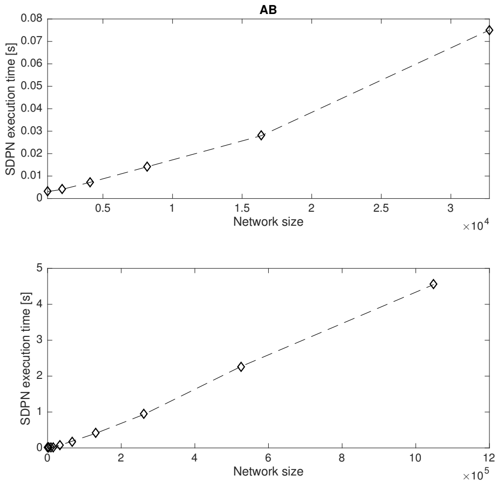

Figure 6: Run-time experiment of the single source mapping algorithm for BarabsiAlbert (BA) model network with sizes from $2^{10} \approx 10^3$ to $2^{20} \approx 10^6$.

References

[1] Agarwal, R., Caesar, M., Godfrey, P. B., and Zhao, B. Y. Shortest paths in less than a millisecond. In *Proceedings of the 2012 ACM Workshop on Workshop on Online Social Networks* (New York, NY, USA, 2012), WOSN '12, ACM, pp. 37–42.

[2] Antulov-Fantulin, N., Lančić, A., Šmuc, T., Štefančić, H., and Šikić, M. Identification of patient zero in static and temporal networks: Robustness and limitations. *Phys. Rev. Lett. 114*, 24 (jun 2015).

[3] Antulov-Fantulin, N., Lancic, A., Stefancic, H., and Sikic, M. Fast-SIR algorithm: A fast algorithm for the simulation of the epidemic spread in large networks by using the susceptible–infected–recovered compartment model. *Information Sciences 239* (2013), 226 – 240.

[4] Boguñá, M., Lafuerza, L. F., Toral, R., and Serrano, M. A. Simulating non-markovian stochastic processes. *Phys. Rev. E 90* (Oct 2014), 042108.

[5] Bortz, A., Kalos, M., and Lebowitz, J. A new algorithm for monte carlo simulation of ising spin systems. *Journal of Computational Physics 17*, 1 (jan 1975), 10–18.

[6] Brockmann, D., and Helbing, D. The Hidden Geometry of Complex, Network-Driven Contagion Phenomena. *Science 342*, 6164 (2013), 1337–1342.

[7] Demetrescu, C., and Italiano, G. F. A new approach to dynamic all pairs shortest paths. *J. ACM 51*, 6 (Nov. 2004), 968–992.

[8] Demetrescu, C., and Italiano, G. F. Algorithmic techniques for maintaining shortest routes in dynamic networks. *Electronic Notes in Theoretical Computer Science 171*, 1 (2007), 3 – 15. Proceedings of the Second Workshop on Cryptography for Ad-hoc Networks (WCAN 2006).

[9] Fichthorn, K. A., and Weinberg, W. H. Theoretical foundations of dynamical monte carlo simulations. *The Journal of Chemical Physics 95*, 2 (1991), 1090.

[10] Kenah, E., and Robins, J. M. Second look at the spread of epidemics on networks. *Phys. Rev. E 76* (2007), 036113.

[11] Lokhov, A. Y., Mézard, M., Ohta, H., and Zdeborová, L. Inferring the origin of an epidemic with a dynamic message-passing algorithm. *Phys. Rev. E 90* (2014), 012801.

[12] Marron, J. S., and Nolan, D. Canonical kernels for density estimation. *Statistics & Probability Letters 7*, 3 (Dec. 1988), 195–199.

[13] Mezard, M., and Montanari, A. *Information, Physics, and Computation.* Oxford University Press, Inc., New York, NY, USA, 2009.

[14] Newman, M. E. J. Spread of epidemic disease on networks. *Physical Review E 66*, 1 (jul 2002).

[15] Pettie, S. A faster all-pairs shortest path algorithm for real-weighted sparse graphs. In *Proceedings of the 29th International Colloquium on Automata, Languages and Programming* (London, UK, UK, 2002), ICALP '02, Springer-Verlag, pp. 85–97.

[16] Thorup, M. *Fully-Dynamic All-Pairs Shortest Paths: Faster and Allowing Negative Cycles.* Springer Berlin Heidelberg, Berlin, Heidelberg, 2004, pp. 384–396.

[17] Tretyakov, K., Armas-Cervantes, A., García-Bañuelos, L., Vilo, J., and Dumas, M. Fast fully dynamic landmark-based estimation of shortest path distances in very large graphs. In *Proceedings of the 20th ACM International Conference on Information and Knowledge Management* (New York, NY, USA, 2011), CIKM '11, ACM, pp. 1785–1794.

[18] Wickramaarachchi, C., Chelmis, C., and Prasanna, V. K. Empowering fast incremental computation over large scale dynamic graphs. In *2015 IEEE International Parallel and Distributed Processing Symposium Workshop* (may 2015), Institute of Electrical and Electronics Engineers (IEEE).

[19] Woolley-Meza, O., Thiemann, C., Grady, D., Lee, J. J., Seebens, H., Blasius, B., and Brockmann, D. Complexity in human transportation networks: a comparative analysis of worldwide air transportation and global cargo-ship movements. *The Eur. Phys. Journal B - Condensed Matter and Complex Systems 84*, 4 (2011), 589–600.
